# Comparative Evaluation of Dentinal Microcrack Formation Following Root Canal Preparation With Three Single-File Nickel-Titanium Systems

**DOI:** 10.7759/cureus.115977

**Published:** 2026-09-08

**Authors:** Mohd Zeeshan Ahmad, Nithin Suvarna, MS Prathap, Diksha Prerna, Kunjam Sawhney, Mallika Rathee

**Affiliations:** 1 Department of Conservative Dentistry and Endodontics, Rama Dental College Hospital and Research Centre, Kanpur, IND; 2 Department of Conservative Dentistry and Endodontics, Yenepoya Dental College, Yenepoya University, Mangalore, IND; 3 Department of Conservative Dentistry and Endodontics, Himachal Institute of Dental Sciences, Paonta Sahib, IND; 4 Department of Conservative Dentistry and Endodontics, Dr. Tooth Dental Studio, Gurugram, IND

**Keywords:** dental cracks, endodontics, micro-computed tomography, nickel-titanium, root canal preparation

## Abstract

Introduction: Dentinal microcracks may develop during mechanical root canal preparation, compromising the structural integrity of root-filled teeth. This study compared dentinal microcrack formation following preparation using three single-file nickel-titanium systems.

Materials and Methods: This in vitro study was conducted on 36 extracted single-rooted mandibular premolars, which were randomly allocated into three groups (n = 12): One-Shape, Flexer ONE, and HyFlex EDM. Micro-computed tomography was used to evaluate dentinal microcracks before and after instrumentation. Incomplete microcracks, complete microcracks, craze lines, and total cracks were assessed. Two-factor mixed analysis of variance with Bonferroni-adjusted post-hoc comparisons was used for statistical analysis, with significance set at p < 0.05.

Results: Instrumentation resulted in a significant increase in incomplete and complete microcracks, craze lines, and total cracks across all three groups (all p < 0.001). The between-group effect was significant for total cracks (p = 0.004), while the group × time interaction was also significant (p = 0.004). Post-hoc analysis demonstrated a significant difference in total crack formation between the One-Shape and HyFlex EDM (mean difference = 2.50, p = 0.002). Postoperatively, One-Shape showed significantly fewer total cracks than Flexer ONE (p = 0.016) and HyFlex EDM (p < 0.001), whereas the difference between Flexer ONE and HyFlex EDM was not significant (p = 1.000).

Conclusion: All three single-file systems increased dentinal microcrack formation after root canal preparation. Although differences were not significant for individual crack categories, the total crack formation differed among the systems, with One-Shape demonstrating the lowest postoperative crack formation.

## Introduction

Root canal preparation is an essential phase of endodontic treatment that aims to eliminate infected or necrotic tissue, facilitate adequate irrigation, and create a suitable shape for three-dimensional obturation [1]. Nickel-titanium (Ni-Ti) rotary and reciprocating instruments have substantially improved the efficiency and predictability of canal preparation owing to their flexibility, cutting efficiency, and ability to maintain the original canal anatomy [2]. The introduction of single-file instrumentation systems has further simplified clinical procedures by reducing the number of instruments required and potentially decreasing instrumentation time [3]. However, the mechanical action of endodontic instruments on dentinal walls may induce structural defects in root dentin [4].

Dentinal microcracks are small defects that can develop or propagate during canal instrumentation. Although their clinical significance remains debated, the progression of dentinal defects may contribute to the development of vertical root fractures and compromise the long-term structural integrity of endodontically treated teeth [5]. The occurrence of these defects can be influenced by several factors, including the instrument design, alloy characteristics, taper, kinematics, cross-sectional configuration, and mechanical stresses generated during instrumentation [6,7]. Therefore, evaluation of microcrack formation is important when assessing the biomechanical safety of different instrumentation systems.

Micro-computed tomography (micro-CT) provides a non-destructive three-dimensional method for evaluating dentinal defects and allows the assessment of crack patterns before and after root canal preparation [8]. Li et al. [9] found that dentinal microcrack lengths increased significantly following root canal preparation, with One-Shape producing greater microcrack alterations than Wave One and Reciproc when assessed using micro-CT.

In addition to instrument design and kinematics, the metallurgical characteristics and mechanical loading behavior of Ni-Ti instruments may influence the stresses transmitted to root dentin during canal preparation. Heat treatment can modify the phase-transformation behavior, flexibility, and mechanical properties of Ni-Ti instruments, thereby altering their interaction with the canal walls and potentially influencing dentinal defect formation [10]. Similarly, torque generation during instrumentation is influenced by factors such as instrument geometry, alloy properties, canal anatomy, and the extent of instrument-dentin engagement. Variations in torque may affect the magnitude and distribution of mechanical stresses transferred to dentin and may therefore contribute to the development of dentinal microcracks [11]. Consideration of these metallurgical and mechanical factors is consequently important when interpreting differences in dentinal microcrack formation among contemporary Ni-Ti instrumentation systems.

Therefore, the aim of the present study was to compare dentinal microcrack formation following root canal preparation using One-shape, Flexer ONE, and HyFlex EDM single-file systems. The primary objective was to evaluate and compare the changes in total dentinal microcracks before and after instrumentation among the three systems, while the secondary objective was to assess and compare the formation of incomplete, complete, and craze lines before and after instrumentation as well as to determine differences among the three experimental groups. The null hypothesis was that root canal preparation would not result in a significant change in dentinal microcrack formation from preoperative to postoperative assessment and that there would be no significant difference in microcrack formation among the three single-file Ni-Ti systems (One-Shape, Flexer ONE, and HyFlex EDM).

## Materials and methods

Study design and setting

This in vitro comparative experimental study was conducted at the Department of Conservative Dentistry and Endodontics, Yenepoya Dental College, India, between May and September 2022. Ethical approval was waived by the Institutional Ethics Committee because the study did not involve human participants or any intervention involving living subjects; only anonymized extracted teeth were used, obtained with appropriate permission for research purposes (YEC2/1058 dated April 20, 2022).

Specimen selection and sample size

Single‑rooted mandibular teeth extracted for orthodontic or periodontal indications were collected and stored in distilled water after disinfection in 0.1% thymol for 24 h at <5°C. The calculus was removed using an ultrasonic scaler, and the external root surfaces were examined under a transilluminated stereomicroscope at ×30 magnification to exclude pre-existing visible cracks. Mesiodistal and buccolingual periapical radiographs were also obtained. The inclusion and exclusion criteria are summarized in Table 1.

**Table 1:** Inclusion and exclusion criteria.

Inclusion criteria	Exclusion criteria
Teeth with a single canal	Teeth with open apices
Teeth with mature apices	Teeth with pulp stones and calcified canals
Straight root canal with <20°curvature	Teeth with dilacerated and resorbed roots
Single apical foramen corresponding to an initial #10 K-file	Teeth with caries, restoration, and surface cracks
Tooth length of 19-22 mm	Teeth with previous root canal treatment

The sample size was calculated a priori using G*Power version 3.1.9.7 (Heinrich Heine University, Düsseldorf, Germany), using an F-test for one-way analysis of variance (ANOVA), an effect size derived from a previous micro-CT study, 80% power, and α = 0.05, resulting in 36 specimens [9]. The teeth were randomly numbered using Random.org and equally allocated into three groups (n = 12): A1, One-Shape; A2, Flexer ONE; and A3, HyFlex EDM.

Experimental procedure

Prior to scanning, the specimens were dried for 2 h at 20°C. Preoperative micro-CT scanning was performed using a SkyScan 1176 system (Bruker MicroCT N.V., Kontich, Belgium) at an isotropic voxel resolution of 17 μm, 80 kV, 313 μA, and a 1-mm aluminum filter. The specimens were positioned perpendicular to the X-ray beam and scanned through 360° with a 0.5° rotation step, 170-ms camera exposure, and frame averaging of 3, requiring approximately 28 min per specimen. Shading correction was applied, and reconstruction was performed using the NRecon software (Bruker MicroCT N.V., Kontich, Belgium) with the Feldkamp cone-beam reconstruction algorithm.

The working length was established 1 mm short of the apical foramen using a #10 K-file (Dentsply Maillefer, Ballaigues, Switzerland), followed by a #15 K-file, to establish the glide path. To simulate periodontal ligament support, the roots were covered with aluminum foil and embedded in a self-curing acrylic resin (Huge™ Self-Curing, Osmunda, Germany), and the resulting space was filled with Flexceed Light-Body vinyl polysiloxane (GC Corporation, Tokyo, Japan).

Root canal preparation was performed by a single calibrated operator under an OPMI pico dental microscope (Carl Zeiss Meditec AG, Jena, Germany) using a Tri Auto ZX2 endodontic motor (J. MORITA MFG. CORP., Kyoto, Japan). All canals were prepared at an apical size of #25. Group A1 was prepared with One-Shape 25/.06 (Micro-Mega, Besançon, France) at 400 rpm and 2.5 N·cm, using outward brushing in the coronal two-thirds and slow in-and-out pecking in the apical third. Group A2 was prepared with Flexer ONE 25/.06 (Mothers International, Adelaide, South Australia, Australia) at 500 rpm and 2.5 N·cm using slow in-and-out pecking. Group A3 was prepared using HyFlex EDM (Coltène/Whaledent AG, Altstätten, Switzerland), with a 10/.05 glide-path file followed by the HyFlex EDM One File to a working length of 500 rpm and 2.5 N·cm.

Irrigation was standardized using 15 mL of 3% sodium hypochlorite per canal with a 30-gauge needle (Ultradent Products Inc., South Jordan, Utah), delivered after every three pecking movements or between the instruments. The final irrigation consisted of 5 mL of 17% ethylenediaminetetraacetic acid, followed by 15 mL of distilled water. After instrumentation, the specimens were rescanned using the same micro-CT parameters and reconstruction protocol as in the preoperative scan.

Microcrack scoring and validation

An original microcrack classification and scoring system was developed in the present study to systematically identify and quantify dentinal defects on micro-CT images (Figure 1).

**Figure 1:**
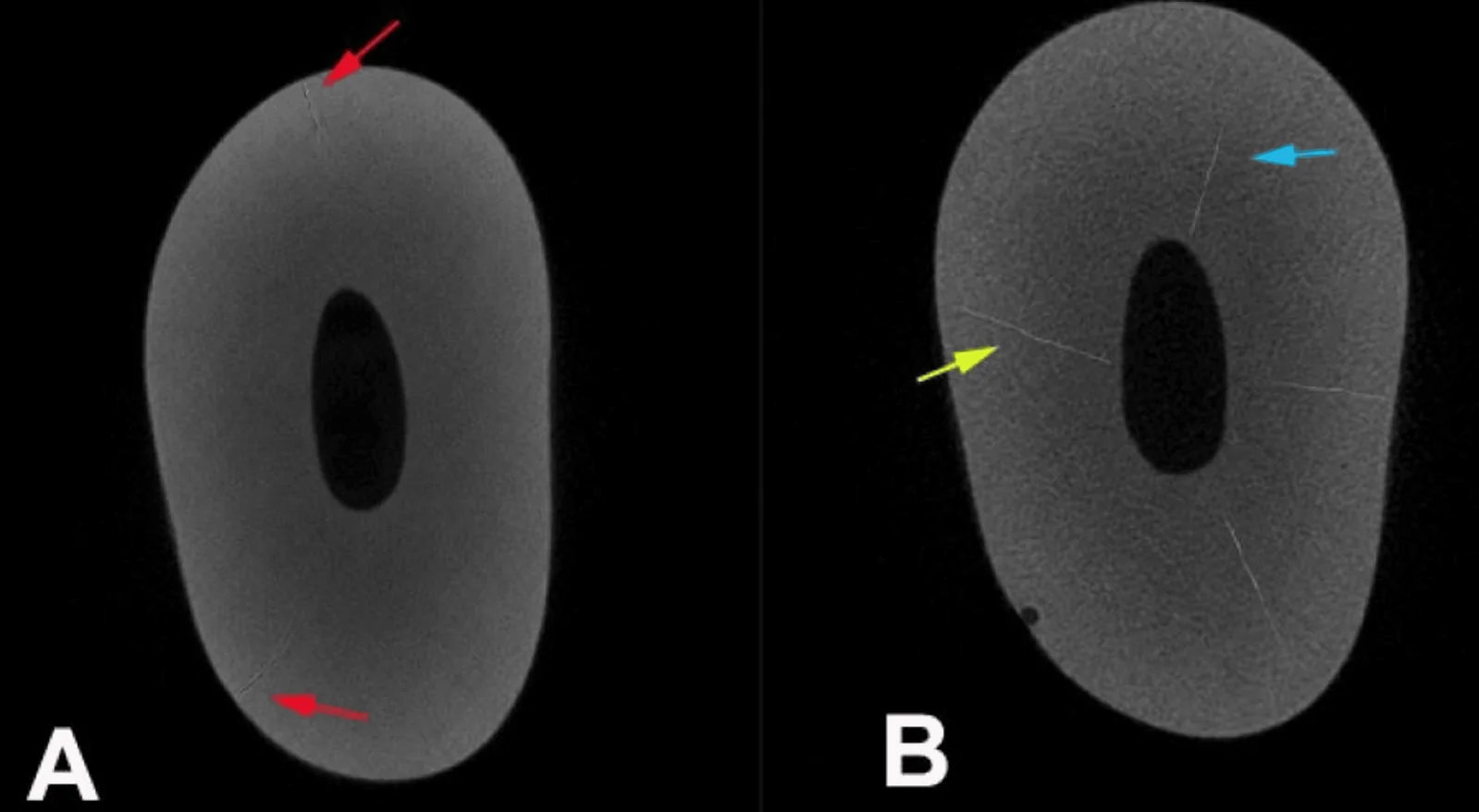
(A) Preoperative micro-computed tomography (micro-CT) image of transverse section of middle third of root showing dentinal craze line (red arrows); (B) Postoperative micro-CT image of transverse section of middle third of root showing complete dentinal crack (yellow arrow) and incomplete dentinal crack (blue arrow). Image acquired from study sample.

Three endodontic specialists (NS, MZA, and MR) independently evaluated the preoperative and postoperative cross-sectional images and classified the defects as incomplete, complete, or craze lines. An incomplete microcrack was defined as a linear defect extending from the root canal wall into the dentin without reaching the external root surface, a complete microcrack extending from the root canal wall to the external root surface, and a craze line as a linear defect extending from the external root surface into the dentin without reaching the canal wall or a line that did not communicate with the canal wall. The total crack count is calculated as the sum of the three categories.

For micro-CT assessment, a continuous crack extending across two or more consecutive slices was considered a single dentinal defect and counted only once, provided that continuity of the same defect could be established across the sequential sections. Separate cracks were counted individually when a distinct interruption or separate crack path was identified. Thus, the unit of scoring was the individual identifiable crack rather than the number of slices through which a crack extended. The preoperative and postoperative micro-CT datasets were registered using the anatomical features of the root to ensure correspondence between the same regions before and after instrumentation. The registered datasets were then compared to identify defects that were newly present after instrumentation. The examiners were blinded to the instrumentation group and assessment status during micro-CT image evaluation to minimize observer bias.

To validate the newly developed classification system, interobserver agreement was assessed using Fleiss’ kappa, with an agreement coefficient of κ = 0.84, whereas intraobserver reliability was evaluated by repeat assessment after four weeks, with a coefficient of κ = 0.82. When a postoperative microcrack was identified, the corresponding preoperative section was reviewed to determine whether it was preexisting or newly developed following instrumentation. In cases of disagreement, the sections were re-examined and discussed by the examiners until a consensus was reached.

Statistical analysis

Statistical analyses were performed using IBM SPSS Statistics for Windows, Version 25.0 (Released 2017, IBM Corp., Armonk, New York). Data are expressed as mean ± standard deviation. A two-factor mixed ANOVA was performed with group as the between-subjects factor and time (preoperative and postoperative) as the within-subjects factor. The main effects of group, time, and the group × time interaction were assessed for incomplete microcracks, complete microcracks, craze lines, and total cracks. Bonferroni-adjusted post-hoc comparisons were performed where appropriate. Statistical significance was set at p < 0.05, with partial eta-squared (η²) and Cohen’s d as effect size measures.

## Results

A total of 36 specimens were included in the final analysis, with 12 specimens assigned to each experimental group. Following instrumentation, the mean numbers of incomplete microcracks, complete microcracks, craze lines, and total cracks increased in all three groups. The increase in total cracks was most pronounced in Group A3 (HyFlex EDM), followed by Group A2 (Flexer ONE) and Group A1 (One-Shape), as shown in Table 2.

**Table 2:** Descriptive analysis of the average number of dentinal microcracks before and after instrumentation. Data are presented as mean ± standard deviation (SD). Group A1: One-Shape; Group A2: Flexer ONE; and Group A3: HyFlex EDM.

Average number of cracks	A1: One-Shape (Mean ± SD)		A2: Flexer ONE (Mean ± SD)		A3: HyFlex EDM (Mean ± SD)	
Crack type	Preoperative	Postoperative	Preoperative	Postoperative	Preoperative	Postoperative
Incomplete microcracks	3.17 ± 0.72	4.33 ± 0.89	3.25 ± 0.75	5.00 ± 1.28	2.92 ± 1.08	5.42 ± 1.38
Complete microcracks	0.67 ± 0.65	1.08 ± 0.67	0.42 ± 0.51	1.17 ± 0.72	0.67 ± 0.49	1.42 ± 0.51
Craze lines	5.67 ± 0.89	6.50 ± 0.67	6.42 ± 0.79	7.67 ± 0.98	6.17 ± 0.83	7.83 ± 1.03
Total cracks	9.50 ± 1.45	11.92 ± 1.00	10.08 ± 0.79	13.83 ± 1.90	9.75 ± 1.48	14.67 ± 1.30

The mixed ANOVA demonstrated a significant effect of assessment time on incomplete microcracks, complete microcracks, craze lines, and total cracks (all p < 0.001), confirming a significant increase in dentinal defects following instrumentation. The effect of instrumentation was not significant for incomplete microcracks (p = 0.389), complete microcracks (p = 0.479), or craze lines (p = 0.053). In contrast, a significant between-group effect was observed for total cracks (F = 6.61, p = 0.004). The Group × time interaction was also significant for total cracks (F = 6.73, p = 0.004), indicating that the change in total crack formation differed according to the instrumentation system (Table 3). These findings indicate that the null hypothesis was rejected, as root canal preparation was associated with a significant increase in dentinal microcrack formation, and significant differences were observed among the instrumentation systems for total crack formation.

**Table 3:** Comparison of dentinal microcrack formation according to instrumentation group and assessment time. A two-factor mixed analysis of variance was used, with the instrumentation group as the between-subjects factor and assessment time as the within-subjects factor. *p < 0.05 is considered statistically significant. The RM factor is repeated measure factor as assessment time. A1: One-Shape; A2: Flexer ONE; A3: HyFlex EDM; η²: Partial eta-squared.

Type of microcracks	Group	Comparison	F value	p-value	η^2^
Incomplete cracks	A1	Preoperative, Postoperative	65.74	< 0.001*	0.42
	A2	Group	0.97	0.389	0.02
	A3	RM Factor x Group	3.00	0.063	0.04
Complete cracks	A1	Preoperative, Postoperative	36.14	< 0.001*	0.23
	A2	Group	0.75	0.479	0.02
	A3	RM Factor x Group	1.09	0.347	0.01
Craze lines	A1	Preoperative, Postoperative	53.67	< 0.001*	0.30
	A2	Group	6.98	0.053	0.15
	A3	RM Factor x Group	1.99	0.153	0.02
Total	A1	Preoperative, Postoperative	176.09	< 0.001*	0.59
	A2	Group	6.61	0.004*	0.07
	A3	RM Factor x Group	6.73	0.004*	0.04

Bonferroni-adjusted post-hoc analysis showed a significant difference in the total crack formation between One-Shape and HyFlex EDM (mean difference = 2.50, p = 0.002), whereas the differences between One-Shape and Flexer ONE (p = 0.140) and between Flexer ONE and HyFlex EDM (p = 0.216) were not significant (Table 4).

**Table 4:** Bonferroni-adjusted pairwise comparison of total dentinal microcrack formation among the three instrumentation groups. Bonferroni-adjusted pairwise comparisons were performed following a two-factor mixed ANOVA. *p < 0.05 was considered statistically significant. A1: One-Shape; A2: Flexer ONE; A3: HyFlex EDM; CI: Confidence interval.

Pairwise comparison	Mean difference	95% CI	Test value	Adjusted p-value	Effect size (Cohen’s d)
A1 vs A2	1.33	−0.34-3.01	2.77	0.140	1.03
A1 vs A3	2.50	0.83-4.17	5.18	0.002*	1.45
A2 vs A3	1.17	−0.51-2.84	2.42	0.216	0.61

The interaction analysis showed no significant differences in the total crack counts among the groups at baseline, indicating comparable preoperative conditions. Following instrumentation, the total crack formation increased significantly in all three groups (p < 0.001). At the postoperative assessment, One-Shape demonstrated significantly fewer total cracks than the Flexer ONE (p = 0.016) and HyFlex EDM (p < 0.001), whereas no significant difference was observed between the Flexer ONE and HyFlex EDM (p = 1.000) (Table 5).

**Table 5:** Post-hoc pairwise comparisons of total dentinal microcrack formation across instrumentation groups and assessment time. The Bonferroni post-hoc test was used for pairwise comparisons of the group × time interaction. *p < 0.05 is considered statistically significant. The total dentinal microcrack score represents the combined score for incomplete microcracks, complete microcracks, and craze lines. A1: One-Shape; A2: Flexer ONE; A3: HyFlex EDM; Pre: Preoperative; Post: Postoperative.

Pairwise comparison	Mean difference	Standard error	t value	Adjusted p-value
Pre-Total A1 - Pre-Total A2	−0.58	0.56	−1.04	1.000
Pre-Total A1 - Pre-Total A3	−0.25	0.56	−0.45	1.000
Pre-Total A1 - Post-Total A1	−2.42	0.56	−4.33	0.001*
Pre-Total A1 - Post-Total A2	−4.33	0.56	−7.76	< 0.001*
Pre-Total A1 - Post-Total A3	−5.17	0.56	−9.25	< 0.001*
Pre-Total A2 - Pre-Total A3	+0.33	0.56	0.60	1.000
Pre-Total A2 - Post-Total A1	−1.84	0.56	−3.28	0.025*
Pre-Total A2 - Post-Total A2	−3.75	0.56	−6.72	< 0.001*
Pre-Total A2 - Post-Total A3	−4.59	0.56	−8.21	< 0.001*
Pre-Total A3 - Post-Total A1	−2.17	0.56	−3.88	0.004*
Pre-Total A3 - Post-Total A2	−4.08	0.56	−7.31	< 0.001*
Pre-Total A3 - Post-Total A3	−4.92	0.56	−8.81	< 0.001*
Post-Total A1 - Post-Total A2	−1.91	0.56	−3.43	0.016*
Post-Total A1 - Post-Total A3	−2.75	0.56	−4.93	< 0.001*
Post-Total A2 - Post-Total A3	−0.84	0.56	−1.49	1.000

Overall, all three single-file systems were associated with a significant increase in dentinal microcracks after instrumentation. However, the magnitude of total crack formation differed significantly among the systems, with One-Shape demonstrating the lowest postoperative crack formation.

## Discussion

The present study demonstrated that root canal instrumentation was associated with a significant increase in incomplete microcracks, complete microcracks, craze lines, and dentinal cracks across all three single-file systems. The significant effect of time indicated that instrumentation contributed to the development or detection of additional dentinal defects. Clinically, this finding reinforces the importance of conservative canal preparation and controlled instrumentation, particularly in teeth with reduced remaining dentinal thickness. However, the in vitro detection of microcracks should not be interpreted as direct evidence of subsequent vertical root fractures.

The increase in the total crack formation was particularly significant, as both the group effect and group × time interaction were significant. HyFlex EDM demonstrated the greatest postoperative increase, whereas One-Shape demonstrated the lowest postoperative total crack count. One possible explanation is the difference in instrument metallurgy, cross-sectional design, taper, and mechanical behavior. HyFlex EDM is manufactured using electrical-discharge machining, which modifies the surface and mechanical characteristics of the instrument. However, its relatively aggressive cutting characteristics and single-file preparation may still generate substantial dentinal stress under particular instrumentation conditions [12,13]. Conversely, the flexibility and controlled cutting behavior of different Ni-Ti systems may influence the magnitude and distribution of the stress transmitted to the canal walls. Previous research has demonstrated that instrument flexibility and alloy characteristics can influence dentinal crack formation [14]. Batra et al. [15] reported that HyFlex EDM produced the fewest dentinal cracks among HyFlex EDM, ProTaper Next, and Wave One Gold, with a significant difference between HyFlex EDM and ProTaper Next in the middle third (p = 0.016); however, no significant differences were observed in the coronal (p = 0.312) or apical thirds (p = 0.076). This contrasts with the present finding of greater crack formation with HyFlex EDM and highlights the influence of methodological factors, such as tooth type, canal anatomy, instrumentation parameters, and crack-detection technique.

Among the three systems, One-Shape demonstrated the lowest total postoperative crack formation rate. One-Shape is a continuously rotating single-file system manufactured from a conventional nickel-titanium alloy and has a 25/.06 preparation design with a variable cross-sectional configuration along its working portion. Its relatively simple single-file sequence may reduce the cumulative mechanical engagement of multiple instruments with the canal wall [15]. In addition, its continuous rotation may provide relatively consistent cutting and debris removal, potentially reducing transient stress accumulation within dentin. The present finding is supported in part by Pedullà et al. [16], who reported that One-Shape did not differ significantly from several other conventional single-file systems with respect to crack formation, although HyFlex EDM and Wave One Gold produced fewer cracks.

Conversely, Liu et al. [17] reported that One-Shape produced root cracks in 7 of 20 teeth (35%), which was significantly higher than the incidence observed with Reciproc (5%) but lower than ProTaper (50%) (p < 0.001). In contrast, no cracks were detected in the Self-Adjusting File or control group. These findings indicate that the propensity for crack formation varies considerably among single-file systems and provide evidence that One-Shape may produce more dentinal defects than some alternative single-file designs. This apparent discrepancy may be attributable to differences in specimen anatomy, file operating parameters, root preparation, and, importantly, crack-detection methodology. Thus, the lower crack formation observed with One-Shape in the present study should be interpreted in the context of specific experimental conditions rather than as evidence that One-Shape is universally less damaging.

The Flexer ONE demonstrated an intermediate increase in the total crack formation. This finding may reflect the balance between cutting efficiency and flexibility, although direct evidence specifically comparing Flexer ONE with One-Shape and HyFlex EDM in relation to dentinal microcrack formation remains limited. Therefore, the intermediate position observed in the present study cannot be attributed to a single design characteristic. Differences in alloy behavior, taper, cross-sectional geometry, instrument-dentin contact, and the amount of dentin engaged during preparation may collectively influence stress transfer to root dentin [4]. This interpretation is consistent with the broader literature, indicating that the mechanical characteristics and flexibility of nickel-titanium instruments can influence dentinal crack formation [6,18]. Pedullà et al. [16] observed differences among single-file systems and suggested that the flexibility associated with heat-treated nickel-titanium alloys may contribute to reduced crack formation. Clinically, the intermediate response of the Flexer ONE suggests that instrument selection should not be based solely on whether a system is classified as a single-file system; rather, its mechanical behavior and interaction with individual canal anatomy should also be considered.

Importantly, the absence of significant between-group differences for incomplete, complete, and craze lines suggests that the apparent differences among the systems were driven predominantly by the overall burden of defects rather than a consistent alteration in a particular crack type. Clinically, this suggests that the selection of a single-file system should not be solely based on the expectation of eliminating dentinal defects. Other factors, including canal anatomy, preservation of dentin, shaping efficiency, instrument separation risk, irrigation, and treatment objectives, should also be considered [3].

Strength of the study

An important methodological strength of the present investigation was the use of preoperative and postoperative micro-CT evaluations. Unlike destructive sectioning, micro-CT permits assessment of the same specimen before and after instrumentation, allowing pre-existing defects to be distinguished from defects identified after preparation [8,19]. This is particularly relevant because pre-existing dentinal defects may otherwise be incorrectly attributed to instrumentation [19]. Furthermore, the present study evaluated the entire root rather than restricting the assessment to predetermined coronal, middle, and apical sections. Consequently, these findings represent the overall burden of dentinal defects rather than their regional distribution. However, differences in voxel resolution, image reconstruction, crack-definition criteria, and thresholding can influence microcrack detection and may partially explain the discrepancies between micro-CT and sectioning studies [20].

Clinical implications

From a clinical perspective, these findings suggest that the instrumentation system may influence the extent of dentinal defect formation, with One-Shape showing the most favorable outcome among the systems evaluated in this study. However, crack formation alone should not determine the clinical file selection. Canal anatomy, preservation of pericervical dentin, shaping efficiency, debris removal, instrument separation resistance, and operator techniques must also be considered. Moreover, the presence of a micro-crack detected by micro-CT does not necessarily indicate that the tooth will subsequently develop a vertical root fracture. Therefore, these findings should be interpreted as evidence of structural alterations rather than direct evidence of clinical failure.

Limitations

The study has several limitations. Its in vitro design cannot fully reproduce the periodontal ligament, occlusal loading, hydration, and biological environment present clinically. The relatively small sample size and restriction to a specific tooth type and canal morphology may also limit the generalizability of the findings. Although specimens were standardized according to predefined anatomical criteria, inherent variations in dentinal structure and canal anatomy may have influenced the mechanical response to instrumentation. Furthermore, instrument-related characteristics, including alloy metallurgy and heat treatment, cross-sectional design, taper, kinematics, and torque generation, may influence the mechanical behaviour of the files and the stresses transmitted to root dentin. These factors were not independently measured or controlled beyond the standardized instrumentation parameters used in the present study and therefore should be considered when interpreting the differences observed among the three systems. In addition, although micro-CT permits non-destructive three-dimensional assessment, its ability to identify very fine defects remains dependent on image resolution and reconstruction parameters. Finally, the study evaluated immediate post-instrumentation changes and therefore could not determine whether the detected defects would propagate under functional loading or ultimately result in vertical root fracture. Further studies incorporating larger samples, clinically representative loading conditions, and direct evaluation of instrument mechanical behaviour are warranted.

## Conclusions

Root canal preparation significantly increased dentinal microcracks across all three single-file systems. Although incomplete microcracks, complete microcracks, and craze lines did not differ significantly among systems, total crack formation demonstrated significant between-group and interaction effects. One-Shape exhibited the lowest postoperative total crack formation under the experimental conditions of this study, whereas HyFlex EDM showed the greatest increase. These findings indicate differences in dentinal defect formation among the tested systems but should not be interpreted as evidence of clinical superiority, particularly given the in vitro design, limited sample size, and potential influence of specimen-related variables. Further clinical and long-term studies are required to determine the clinical significance of these findings.

## References

[1] Gomes BP, Aveiro E, Kishen A (2023). Irrigants and irrigation activation systems in endodontics. Braz Dent J.

[2] Singh A, Saha S, Dhinsa K (2025). Revolutionizing pediatric endodontics: a two-decade narrative on the evolution, efficiency, and future of pediatric rotary systems. Cureus.

[3] Srivastava S (2024). Root canal instrumentation: current trends and future perspectives. Cureus.

[4] Iosif L, Dimitriu B, Niţoi DF, Amza O (2023). Endodontic dentistry: analysis of dentinal stress and strain development during shaping of curved root canals. Healthcare (Basel).

[5] Aksoy Ç, Keriş EY, Yaman SD, Ocak M, Geneci F, Çelik HH (2019). Evaluation of XP-endo Shaper, Reciproc Blue, and ProTaper Universal NiTi Systems on dentinal microcrack formation using micro-computed tomography. J Endod.

[6] Ustun Y, Aslan T, Sagsen B, Kesim B (2015). The effects of different nickel-titanium instruments on dentinal microcrack formations during root canal preparation. Eur J Dent.

[7] Shemesh H, Bier CA, Wu MK, Tanomaru-Filho M, Wesselink PR (2009). The effects of canal preparation and filling on the incidence of dentinal defects. Int Endod J.

[8] Bayram HM, Bayram E, Ocak M, Uzuner MB, Geneci F, Celik HH (2017). Micro-computed tomographic evaluation of dentinal microcrack formation after using new heat-treated nickel-titanium systems. J Endod.

[9] Li ML, Liao WL, Cai HX (2018). A micro-computed tomographic evaluation of dentinal microcrack alterations during root canal preparation using single-file Ni-Ti systems. Exp Ther Med.

[10] Kwak SW, Shen Y, Liu H, Wang Z, Kim H-C and Haapasalo M (2021). Heat treatment and surface treatment of nickel-titanium endodontic instruments. Front Dent Med.

[11] Kwak SW, Shen Y, Liu H, Kim HC, Haapasalo M (2022). Torque generation of the endodontic instruments: a narrative review. Materials (Basel).

[12] Pirani C, Iacono F, Generali L (2016). HyFlex EDM: superficial features, metallurgical analysis and fatigue resistance of innovative electro discharge machined NiTi rotary instruments. Int Endod J.

[13] Kimura S, Ebihara A, Maki K, Kyaw MS, Kasuga Y, Omori S, Okiji T (2024). Phase transformation behavior and mechanical properties of HyFlex EDM nickel-titanium endodontic rotary instrument: evaluation at body temperature. J Dent Sci.

[14] Kim HC, Lee MH, Yum J, Versluis A, Lee CJ, Kim BM (2010). Potential relationship between design of nickel-titanium rotary instruments and vertical root fracture. J Endod.

[15] Batra R, Dixit A, Tiwari A, Kumar A, Sinha S, Badnaware S, Singh R (2023). Comparative evaluation of dentinal defects after root canal preparation using various nickel titanium files: an in vitro study. Cureus.

[16] Pedullà E, Genovesi F, Rapisarda S, La Rosa GR, Grande NM, Plotino G, Adorno CG (2017). Effects of 6 single-file systems on dentinal crack formation. J Endod.

[17] Liu R, Hou BX, Wesselink PR, Wu MK, Shemesh H (2013). The incidence of root microcracks caused by 3 different single-file systems versus the ProTaper system. J Endod.

[18] Çitak M, Özyürek T (2017). Effect of different nickel-titanium rotary files on dentinal crack formation during retreatment procedure. J Dent Res Dent Clin Dent Prospects.

[19] De-Deus G, Silva EJ, Marins J (2014). Lack of causal relationship between dentinal microcracks and root canal preparation with reciprocation systems. J Endod.

[20] Pinto JC, Coaguila-Llerena H, Torres FF, Lucas-Oliveira É, Bonagamba TJ, Guerreiro-Tanomaru JM, Tanomaru-Filho M (2021). Influence of voxel size on dentinal microcrack detection by micro-CT after root canal preparation. Braz Oral Res.

